# Sunitinib effectively reduces clonogenic acute myeloid leukemia cells *in vitro*

**DOI:** 10.1186/1471-2164-15-S2-P67

**Published:** 2014-04-02

**Authors:** Farid Ahmed, Manal Al-Oteibi, Samah Layati, Fatima Kadi, Adeel Chaudhary, Mamdooh Gari, Mohammed Al-Qahtani

**Affiliations:** 1 Center of Excellence in Genomic Medicine Research, King Abdulaziz University, Jeddah, Saudi Arabia

## Background

Acute myeloid leukemia (AML) is a clonal hematopoietic disorder characterized by enhanced proliferation and block in differentiation of immature myeloid cells. AML pathogenesis is attributed to several recurrent chromosomal abnormalities in nearly half of the AML cases while the rest of the AML cases show at least one or more molecular abnormalities. Activating mutations and/or high expression of receptor tyrosine kinase (RTK) family members (FLT3, cKIT) have been well documented in AML. Specific RTK inhibitors (TKI) have been developed for targeted therapy of AML and many are currently under study [1]. Sunitinib is a potent multi-TKI that has been FDA approved for renal carcinoma and imatinib resistant gastro intestinal tumors. Although some studies show the effect of Sunitinib on AML cell line growth, there has been no investigation of its effect on AML clonogenic cells [2]. In the current study, we investigated the effect of Sunitinib on clonogenic AML cells and normal hematopoietic progenitors *in vitro*.

## Materials and methods

Human primary AML patient bone marrow or peripheral blood mononuclear cells (AML-MNC) were isolated by density gradient centrifugation. Human normal hematopoietic stem/progenitors cells (HSPC) were isolated from umbilical cord blood using anti-CD34 antibodies on a magnetic separator (Miltenyi Biotech). AML-MNCs and HSPC were incubated in SFEM (Stem Cell Technologies) with varying concentrations of Sunitinib malate (Sigma) and DMSO as vehicle control for 24 hours. Clonogenic assays were performed by plating the drug treated cells in Methycellulose and the colonies were evaluated after 10-14 days. CD34^+^ ALDH^+^ cells were stained using Aldefluor Kit (Stem Cell Technologies) and CD34-VioBlue antibodies (Miltenyi Biotech).

## Results

**Figure 1:**
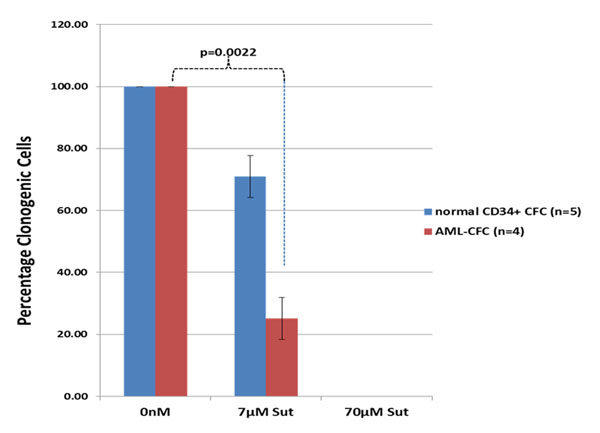
Inhibition of AML-CFC (n=4) and normal CD34^+^ -cell derived CFC with Sunitinib.

With 7µM Sunitinib, there was a 75% reduction (±6.79%; n=4) of AML-CFC as compared to untreated, while 70µM Sunitinib treatment showed total eradication of AML-CFC. Treatment of normal HSPC with 7µM Sunitinib showed only 29% reduction (±6.77%; n=5) (Figure 1) of normal CFC. In two additional AML cases, CD34^+^ALDH^+^cells and CD34^-^ ALDH^-^ cells were sorted and incubated with or without Sunitinib. AML colonies were detected only in CD34^+^ALDH^+^ population that showed total inhibition by 7 µM Sunitinib.

## Conclusions

Sunitinib is effective in reduction of clonogenic AML cells. Sunitinib also inhibits normal HSPC derived CFCs albeit less effectively than AML-CFC. This study highlights the relevance of TKI inhibitors in targeting AML. Further studies are aimed at confirming the molecular mechanisms involved in the inhibition as well as taking this study further for targeting patient derived leukemic stem cells using immuno-deficient mouse models.

*Authors would like to thank KACST for funding this project* (*grant number 09BIO-693-03*)*.*
